# The relationship of smoking to cg05575921 methylation in blood and saliva DNA samples from several studies

**DOI:** 10.1038/s41598-021-01088-7

**Published:** 2021-11-03

**Authors:** Kelsey Dawes, Allan Andersen, Rachel Reimer, James A. Mills, Eric Hoffman, Jeffrey D. Long, Shelly Miller, Robert Philibert

**Affiliations:** 1 Department of Psychiatry, University of Iowa, Iowa City, IA 52242 USA; 2 Molecular Medicine Program, University of Iowa, Iowa City, IA 52242 USA; 3 Department of Public Health, Des Moines University, Des Moines, IA 50312 USA; 4 Department of Radiology, University of Iowa, Iowa City, IA 52242 USA; 5 Department of Biomedical Engineering, University of Iowa, Iowa City, IA 52242 USA; 6 Department of Biostatistics, University of Iowa, Iowa City, IA 52242 USA; 7 Behavioral Diagnostics LLC, Coralville, IA 52241 USA

**Keywords:** Diagnostic markers, DNA methylation

## Abstract

Numerous studies have shown that cg05575921 methylation decreases in response to smoking. However, secondary to methodological issues, the magnitude and dose dependency of that response is as of yet unclear. This lack of certainty is a barrier to the use of DNA methylation clinically to assess and monitor smoking status. To better define this relationship, we conducted a joint analysis of methylation sensitive PCR digital (MSdPCR) assessments of cg05575921 methylation in whole blood and/or saliva DNA to smoking using samples from 421 smokers and 423 biochemically confirmed non-smokers from 4 previously published studies. We found that cg05575921 methylation manifested a curvilinear dose dependent decrease in response to increasing cigarette consumption. In whole blood DNA, the Receiver Operating Characteristic (ROC) Area Under the Curve (AUC) of cg05575921 methylation for predicting daily smoking status was 0.98. In saliva DNA, the gross AUC was 0.91 with correction for cellular heterogeneity improving the AUC to 0.94. Methylation status was significantly associated with the Fagerstrom Test for Nicotine Dependence score, but with significant sampling heterogeneity. We conclude that MSdPCR assessments of cg05575921 methylation are a potentially powerful, clinically implementable tool for the assessment and management of smoking.

## Introduction

Despite sustained efforts, smoking continues to be one of the largest preventable cause of premature death in the world^1^. A variety of pharmacological and behavioral treatments for smoking exist^2^. But in actual clinical practice, these measures have unacceptably high failure rates^2^. Although there are a number of reasons for these high failure rates, one of the potentially more addressable reasons may be weaknesses in current biomarker approaches for quantifying smoking and monitoring smoking cessation.

Currently, two biomarkers are used clinically to guide the assessment and treatment of smoking^3^. The first is carbon monoxide (CO). CO measurements are easy to perform. However, the short half-life of CO prevents it from being used to assess daily levels of cigarette consumption and makes it insensitive to low intensity or sporadic smoking^3,4^. Cotinine assessments are more sensitive and can be used to more effectively gauge the level of cigarette consumption^3,5^. But the use of nicotine replacement therapy and the use of non-combustible products has rendered this valuable test less informative. Conceivably, a test that could be conducted in standard clinical settings and that could quantitate cigarette consumption even in the face of other forms of nicotine consumption could find clinical utility.

DNA methylation testing may be that new acceptable form of testing. Since 2008, scores of studies have examined the relationship of smoking to DNA methylation status^6^. In general, these studies have shown that smoking is associated with broad changes across the epigenome with the majority of these changes at least partially reverting in response to smoking cessation^7–9^. Using this methylation information, several groups have attempted to construct methylation indices for the prediction of smoking^5,10,11^. In general, these indices have worked well but have two substantial failings. First of all, many of these indices were constructed using samples from subjects whose smoking status was not biochemically verified. This is a concern because we have repeatedly shown that substantial numbers of subjects participating in research studies (4–10%) who deny any lifetime use of nicotine products have significant serum levels of cotinine^12–14^. Second, these indices rely on array hybridization techniques, which are costly, relatively imprecise, time consuming and reference dependent that do not produce reliable beta values for clinical interpretation^15–17^.

Precision Epigenetics methods targeting highly predictive loci may be a better approach. In particular, measurements of cg05575921, a CpG residue contained within an intronic transcription enhancer in the *aryl hydrocarbon receptor repressor* (AHRR) gene, may have considerable potential for clinical use^18,19^. Genome wide studies of smoking consistently show that it is the locus most highly associated with smoking in individuals of European, African and Asian ancestries with the difference in blood DNA methylation between smokers and non-smokers (i.e. ∆β) typically being between 15 and 25%^19–21^. Most critically, as opposed to other single and multipoint methods for assessing methylation status, ethnic specific variation does not affect the methylation set point of cg05575921^22^.

In 2018, we introduced a methylation sensitive digital PCR (MSdPCR) assay for assessing methylation status at cg05575921. The assay, which is configured to run on the FDA approved Bio-Rad QX200 platform, has inter-assay variation of 0.7% and can be completed in approximately 4 to 5 h^23^. Most importantly, unlike quantitative PCR tests whose determinations are dependent upon reference standards, digital PCR tests are reference/calibration-free methods with clearly established metrics for precision^23–25^. This allows the results from different runs or labs to be compared without need to control for batch variation. As such, if cg05575921 methylation can be used to predict smoking, this MSdPCR test theoretically has many of the characteristics of an ideal assay for Precision Epigenetic approaches for assessing smoking.

Over the past 3 years, we have used this assay to assess cg05575921 methylation in the DNA prepared from the whole blood (WB) of four populations of subjects who were clinically characterized for smoking status and with the non-use status of all control subjects being verified using both serum cotinine and exhaled carbon monoxide assessments^26–29^. In an attempt to provide a more robust understanding of the relationship of WB cg05575921 methylation to smoking status, we now jointly analyze those results. In addition, we introduce the methylation assessments of DNA from the paired saliva samples for many of those samples and conduct parallel analyses of those results.

## Methods

The data for these analyses are derived from subjects included in four previously published studies of smoking or alcohol use^26–29^. In each of these studies, methylation status at cg05575921 status in WB or saliva DNA was determined using MSdPCR and the smoking status of each non-smoking control was verified using serum cotinine assessments and carbon monoxide assessments as previously described^26,29^. All procedures were performed in accordance with the Declaration of Helsinki with the individual institutional approvals noted separately for each cohort being noted in the supplementary methods section with each subject providing written informed consent.

The Nicotine Cessation (NC) cohort subjects were collected as part of a project whose goal was to help define the reversion curve of cg05575921 in response to smoking cessation^26^. In brief, subjects from an Iowa community substance use treatment who stated an interest in smoking cessation, reported smoking at least 8 cigarettes per day and had an expired carbon monoxide level of 8 ppm or greater, were eligible for inclusion for this longitudinal study of smoking cessation. After consent, adult subjects were interviewed with a modified version of the Semi-Structured Assessment for the Genetic of Alcoholism, Ver 2 (SSAGA-II) and the Substance Use Questionnaire^30,31^. Then, both WB and saliva specimens were collected to provide biomaterials for serum and DNA preparation. A total of 114 subjects enrolled in the study and completed the intake visit.

The Alcohol Cessation (ALC2A) subjects were collected for a project whose goal was to develop a blood-based test for heavy alcohol consumption^27^. In this protocol, two distinct groups of subjects were collected. The first group consisted of self-reported smokers who were admitted to one of three Iowa alcohol treatment centers for the treatment of severe alcohol use disorder in the context of current intoxication. The second group of subjects (i.e. controls) collected under this award were individuals solicited from the University of Iowa community who denied any history of substance abuse and reported at least one year of abstinence from alcohol. After informed consent was received, both sets of subjects (127 smokers and 144 controls) were then were interviewed with a modified version of the SSAGA-II and the Substance Use Questionnaire, with both saliva and blood collected for biomaterial preparation^30,31^.

The Smoke Free World (SFW) cohort was collected as part of a project to understand whether DNA methylation could differentiate those who use combustible forms of tobacco from those who use other nicotine containing products^29^. After providing informed consent, adult subjects were administered a 320 item REDCap® interview that assessed nicotine product use history, then phlebotomized and saliva sampled to provide biomaterials for the current study^29,32^. Only the data from the smoking subjects and the lifetime non-smoking controls were included in this study.

The Smoking Computerized Tomography (SCT) cohort was collected as part of a project whose purpose was to understand the relationship of sildenafil on pulmonary^28^. In this protocol, subjects who smoked at least 10 cigarettes per day and had a 5-pack year history of smoking were invited to complete a REDCap® administered prescreening interview to assess smoking history and study eligibility. After receiving informed consent, subjects were phlebotomized to provide blood for DNA for epigenetic analysis and serum.

Serum cotinine values presented herein for the SFW cohort were determined as previously described using enzyme-linked immunoassay reagents from AbNova (Taiwan) and a Molecular Devices Emax spectrophotometer (Sunnydale). Fagerstrom Test for Nicotine Dependence (FTND) scores for three of the cohorts (NC, AlcC and SCT) was assessed and scored as previously described^33^. Exhaled carbon monoxide levels were assessed using a Smokelyzer® according to the manufacturer’s protocol (CoVita, USA). All control subjects included in this study had serum cotinine values < 2 ng/ml and exhaled carbon monoxide levels of < 8 parts per million.

DNA from WB and saliva was prepared as previously described^26^. DNA methylation at cg05575921 and DMR11 for both WB and saliva DNA was determined as previously described. In brief, 1 µg of DNA of either whole blood or saliva DNA was bisulfite converted using a EpiTect® Fast DNA kit from Qiagen (Germany) according to manufacturer’s directions. An aliquot of each bisulfite-converted sample was pre-amplified, diluted 1:3000 with molecular grade water, and partitioned into ~ 1.5 nl droplets using an automated droplet generator. DNA amplicons contained within these droplets were then PCR amplified using proprietary primer probe sets (Smoke Signature® or DMR11) for each locus from Behavioral Diagnostics (Coralville, IA) and universal digital PCR reagents from Bio-Rad (Carslbad, CA). The number of droplets containing amplicons with at least one “C” allele (methylated CpG residue), one “T” allele (unmethylated CpG residue) or neither allele was then determined using Bio-Rad QX-200 droplet reader. Percent methylation was calculated using Quantisoft software by fitting the observed ratios to a Poisson distribution. Please also note that because MSdPCR is a reference free method, correction for batch variation is not necessary^24,34^.

The relative contribution of leukocyte DNA (X) to the total DNA sample was determined using the following equation: ${DMR11}^{obs}=\left[0.01X+0.99\left(1-X\right)\right]$ where DMR11^obs^ is the methylation status at DMR11 in the saliva sample, and 0.01 and 0.99 are the fractional methylation values of DMR11 in leukocytes and buccal cells, respectively^35^.

Data Analyses: In general, statistical analyses were conducted with R statistical software (v 1.3.959), and JMP Version 10 (SAS Institute, Cary, NC). Linear regression was used to examine the relationships between cg05575921 methylation, demographic variables, FTND, and self-reported cigarette consumption. All reported R^2^ values are adjusted for the sample size and number of features. The significance of differences in group means was assessed using Welch’s Two Sample t-tests^36^. Smoothing splines (λ = 100,000) were fit in JMP to examine the nonlinear relationship of cg05575921 methylation to self-reported cigarette consumption^37^.

Models for predicting smoking status were generated separately using whole blood (WB, Model 1) and saliva (Model 2) DNA data. The first goal of each model building exercise was to examine the extent to which abstaining from smoking was predicted in a similar manner among the four studies. The second goal was to combine the data from all the studies and estimate general models for predicting smoking abstinence. Cox testing was performed to compare linear regression models using the *coxtest* function in the *lmtest* R package^38^.

For all analysis, logistic regression was used with the binary response being abstinence (0 = smoker, 1 = control)^39^. A complication was that NC and SCT cohorts had only smokers, and logistic regression models could not be estimated. Therefore, we trained regression models separately on ALC2A and SFW and tested them on all the data sets individually. Predictive performance was evaluated with the Brier score, as this can be computed regardless if a data set has only smokers, or a mix of smokers and controls^40^. The Brier score is defined as $Brier= \frac{1}{N}\sum {\left({p}_{i}-{o}_{i}\right)}^{2}$, where ${p}_{i}$ is the predicted probability for the ${i}$th participant and ${o}_{i}$ is the observed value (0 or 1)^40,41^.

Two training models were considered, one for WB cg05575921(Model 1) and the other for saliva cg05575921 (Model 2). Model 1 had the single predictor of WB cg05575921 methylation (%). Model 2 had uncorrected saliva cg05575921 methylation (%), DMR11 methylation (%), and their interaction (product term).Testing consisted of using the estimated coefficients of a training model to compute the Brier score for ALC2A, SFW, NC, SCT (note that the training and test sets were sometimes identical). An additional complication was that the SCT cohort did not have saliva cg05575921 and thus, was excluded from the Model 2 testing. Confidence intervals were computed using the non-parametric bootstrap^42^.

### Ethics approval and consent to participate

All procedures were performed in accordance with the Declaration of Helsinki. Institutional Review Board (IRB) approvals were provided by the University of Iowa IRB (IRB 201905678 and IRB201706713) and the Western Institutional Review Board (WIRB 20160135 and WIRB20162083). All subjects in each of the cohorts provided written informed consent for the procedures.

## Results

### Clinical demographics

The key demographic and tobacco use characteristics of the subjects from each of the four cohorts are given in Table 1. In total, the four cohorts contain 421 smoking and 423 non-smoking subjects (*N* = 843) with 603 subjects having both whole blood and saliva DNA methylation data.

**Table 1:** Key clinical characteristics of subjects in each cohort.

Cohort	Group	Gender	N	Age	Cigs/Day	Cg05575921
ALC2A	Smoker	Male	84	41 ± 11.7	19.4 ± 13.8	48.9% ± 18.0
	Smoker	Female	43	40.8 ± 11.3	14.2 ± 9	49.6% ± 21.0
SFW	Smoker	Male	45	42.8 ± 14.9	16.3 ± 9.6	53.7% ± 17.8
	Smoker	Female	67	40.1 ± 11.5	14.5 ± 7.9	55.2% ± 20.1
SCT	Smoker	Male	35	42.5 ± 10.9	19.9 ± 10.3	47.0% ± 17.6
	Smoker	Female	32	45.1 ± 8.9	14.7 ± 4.2	57.3% ± 19.0
NC	Smoker	Male	70	42.3 ± 11.7	14.1 ± 8.3	51.4% ± 14.2
	Smoker	Female	45	41.5 ± 13.5	12.7 ± 7.5	52.0% ± 15.8
Total		Male	234	41.9 ± 12.1	17.3 ± 11.3	50.2% ± 16.8
		Female	187	41.5 ± 11.6	14.1 ± 7.6	53.6% ± 19.2
ALC2A	Control	Male	68	40.2 ± 14.6	–	85.6% ± 3.4
	Control	Female	86	42.2 ± 14.8	–	86.6% ± 2.3
SFW	Control	Male	80	30.3 ± 12.8	–	86.0% ± 3.3
	Control	Female	189	30.4 ± 11.4	–	87.1% ± 2.5
Total		Male	148	34.9 ± 14.5	–	85.8% ± 3.3
		Female	275	34.1 ± 13.6	–	87.0% ± 2.5

Consistent with the overall distribution in the population, the majority of the smoking subjects were male (55%, 234 of 421). In contrast, the majority of the non-smoking controls were female (65%, 275 of 423).

Smokers in each of the four cohorts tended to be in their early to mid 40’s with an average age of 41.9 ± 12.1 and 41.5 ± 11.6 for males and females, respectively. The control subjects tended to be younger with both the male (34.9 ± 14.5 vs 41.9 ± 12.1, *p* < 0.0001) and the female (34.1 ± 14.6 vs 41.5 ± 11.6) subjects being significantly younger than their corresponding smoking counterparts.

The smoking subjects reported smoking as little as one cigarette and as much as 60 cigarettes per day. But overall, they reported consuming approximately ¾ of a pack daily over the past month with males smoking significantly more than females (17.3 ± 11.3 vs 14.1 ± 12.1, *p* < 0.001).

**Figure 1:**
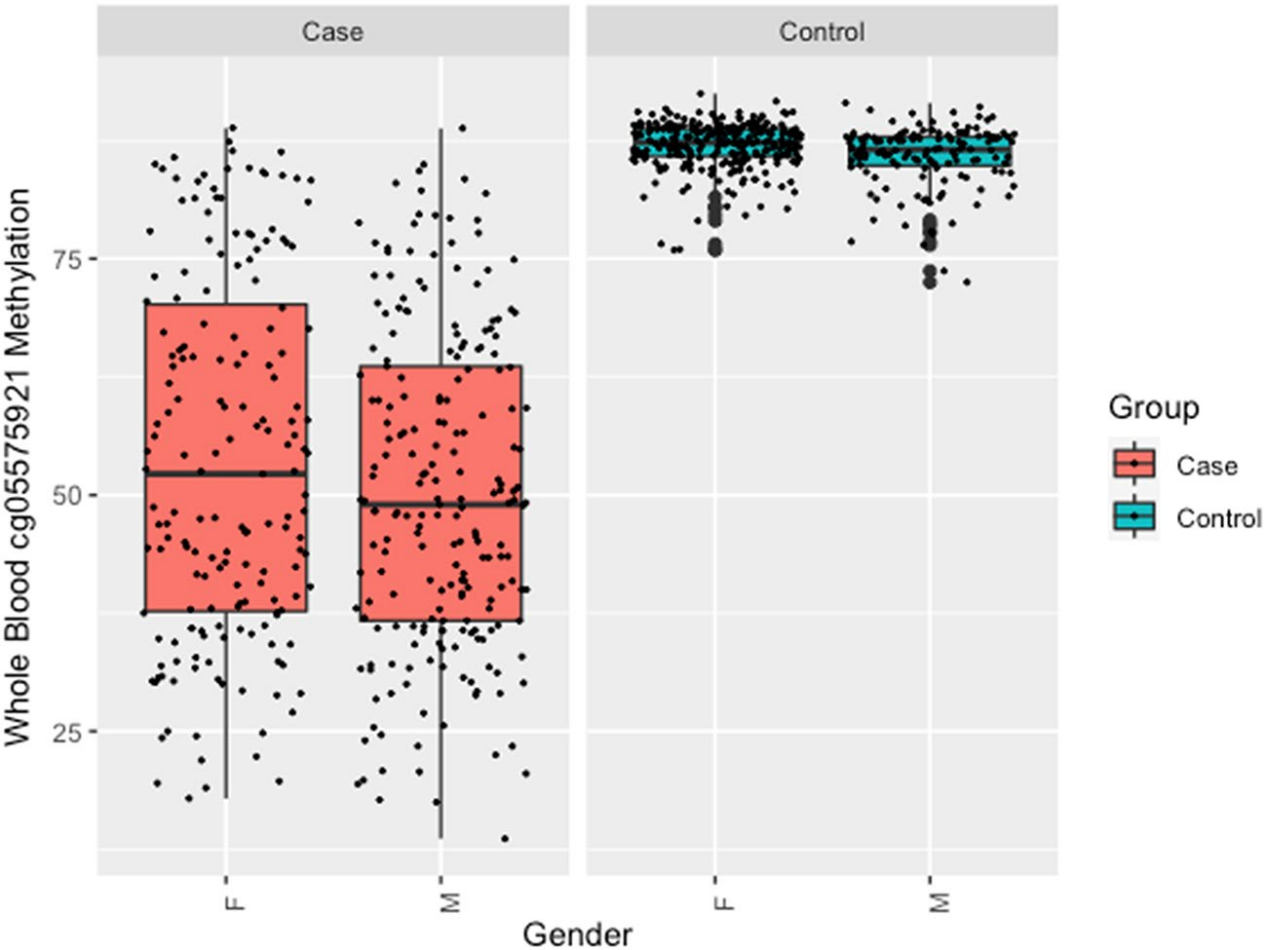
Box plots of the distribution of cg05575921 values in WB samples from case (n = 421) and control (n = 423) subjects as a function of gender (M = male, F = female).

There was no significant difference between the WB cg05575921 levels of male and female smokers (50.3% ± 16.8 vs 53.6% ± 19.2, *p* < 0.685). Although the range of values was much more restricted (see Fig. 1), female non-smokers had a slight, but significantly higher levels of cg05575921 methylation than male non-smokers (87.0% vs 85.8%, *p* < 0.0001).

Consistent with the localities in which the subjects were ascertained, the majority of subjects in the study were White, with 84% for smokers and 88% for controls. However, there was significant representation of other ethnicities with 11% of the smoking subjects being African American and 3% for controls. Asians constituted 1% of smokers and 6% of controls (“other” ethnic categories constituted 4% of smokers and of controls).

#### Other biomarker status

We next examined the relationship of WB cg05575921 methylation to serum cotinine (COT) and exhaled carbon monoxide (CO) levels. Because the ALC2A, NC and SCT cohorts were ascertained as part of smoking cessation protocols with a number of the ALC2A subjects on the nicotine patch, the serum cotinine levels of these subjects may not represent steady state levels of cotinine. However, the SFW subjects were not ascertained as part of a treatment program. In these SFW subjects, serum cotinine values were highly correlated with cg05575921 methylation (Supplemental Fig. 1; r = − 0.82, *p* < 2.2e−16). Similarly, CO was also highly correlated (Supplemental Fig. 2; r = − 0.74, *p* < 2.2e−16).

**Figure 2:**
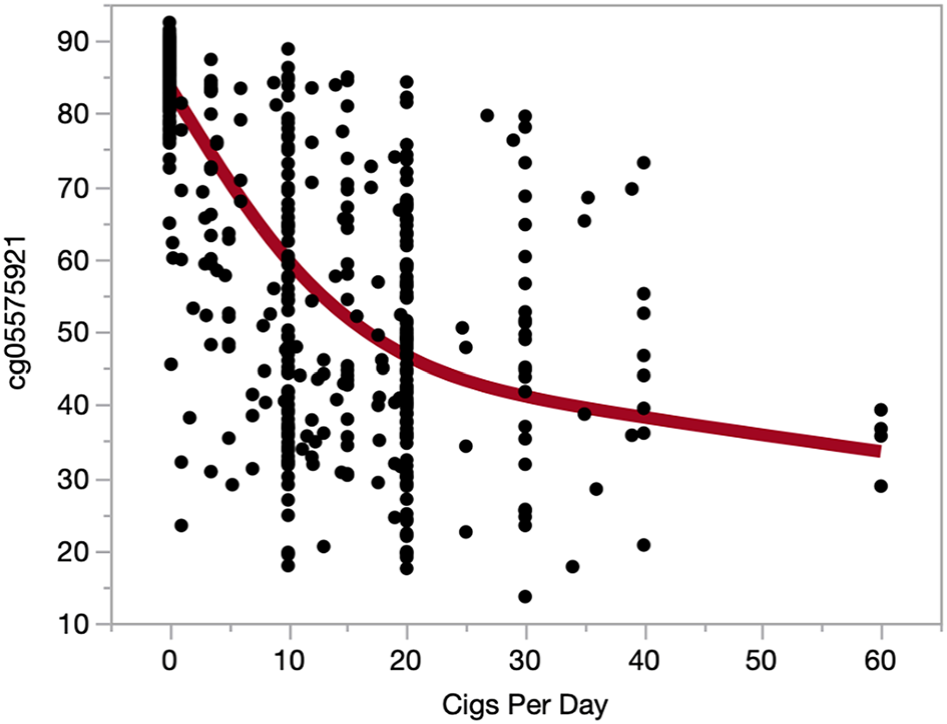
The relationship of WB cg05575921 methylation to self-reported daily cigarette consumption. The line in red represent a smoothing spline fit with a lambda of 100,000 (*p* < 0.0001, R^2^ = 0.6446).

#### Consumption and dependence variables

**Figure 3:**
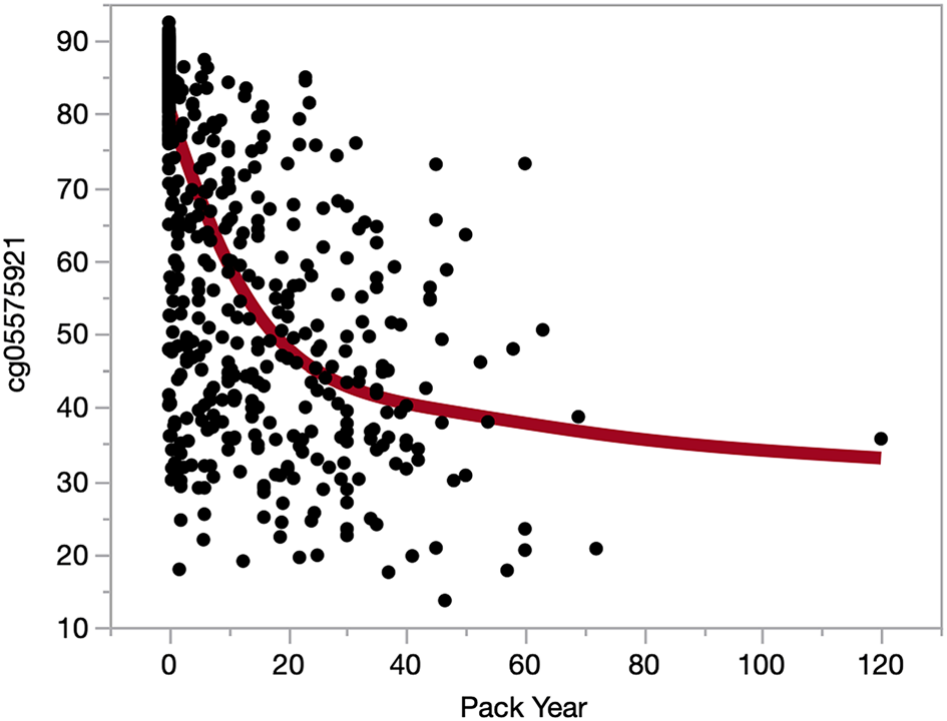
The relationship of WB cg05575921 methylation to lifetime cigarette consumption expressed as pack years. The line in red represent a smoothing spline fit with a lambda of 100,000 (*p* < 0.0001, R^2^ = 0.5365).

Next, we examined the ability of WB cg05575921 methylation to predict average daily cigarette consumption within the past month. Using a smoothing spline, a significant curvilinear dose response is seen (Fig. 2; *p* < 0.0001, R^2^ = 0.6446). A sharp drop in methylation with increasing levels of daily cigarette consumption, with a more gradual decline in methylation seen after ~ 20 cigarettes per day. We also examined the relationship of pack year history to cg05575921 methylation status. Using a smoothing spline, a significant non-linear dose response is seen (Fig. 3; *p* < 0.0001, R^2^ = 0.5365).

In pooled case subjects, FTND was negatively correlated with both WB cg05575921 methylation (*p* = 2.64e−05, R^2^ = 0.0612), and average self-reported cigarettes consumed per month (*p* = 0.006, R^2^ = 0.023). Age, sex and ethnicity were not significantly associated with FTND. Cox Testing demonstrated a significant effect of group status on the ability of cg05575921 to predict FTND (*p* < 0.0001). In the ALC2A and NC case subjects, FTND did not have a significant relationship with WB cg05575921 methylation, cigarettes consumed per month or key demographics. But in the SCT case subjects, significant relationships between FTND and WB cg05575921 methylation (*p* = 1.28e−04, R^2^ = 0.1966), self-reported average cigarettes consumed per month (*p* = 1.84e−05. R^2^ = 0.2392), and male gender (*p* = 0.0425, R^2^ = 0.04811) were observed. The SFW case subjects did not have FTND data available.

#### Prediction capacity

For the final set of analyses of the WB data, logistic regression was used to examine the ability of WB cg05575921 methylation to predict current smoking status. Table 2 shows the Brier score results of the training and testing sets for Model 1. The upper portion shows the Brier score and 95% CI when training on either ALC2A or SFW (left) and testing on the four data sets (top). The bold entries indicate that the testing and training data sets were identical. The first row shows that when the model is trained on ALC2A, the Brier score is slightly larger when testing on SFW and SCT than when testing on ALC2A itself, but slightly smaller when testing on NC. The second row shows that when the model is trained on SFW, the Brier score is larger when testing on SCT than testing on SFW itself, but slightly smaller when testing on ALC2A and NC. The lower portion of Table 3 shows the difference between the Brier score trained and tested on the same data set (bold entries in the upper table) and tested on a different data set. For ALC2A, all the 95% CIs for the difference contain 0. For SFW, the only difference for which the CI does not contain 0 is SFW-SFW vs SFW-ALC2A, indicating a smaller Brier score for the latter.

**Table 2:** Model 1 (WB DNA) Brier scores and Brier scores differences for the test and training data sets.

Train	Test			
	ALC2A	SFW	NC	SCT
**Brier score**				
ALC2A	**0.0421 (0.0410, 0.0478)**	0.0493 (0.0468, 0.0580)	0.0340 (0.0211, 0.0528)	0.0705 (0.0509, 0.1000)
SFW	0.0423 (0.0410, 0.0470)	**0.0480 (0.0468, 0.0536)**	0.0372 (0.0239, 0.0533)	0.0740 (0.0536, 0.1021)
**Difference**				
ALC2A	–	− 0.0072 (− 0.0139, − 0.0023)	0.0081 (− 0.0056, 0.0195)	− 0.0284 (− 0.0496, − 0.0127)
SFW	0.0057 (0.0004, 0.0116)	–	0.0108 (− 0.0058, 0.0249)	− 0.0260 (− 0.0540, − 0.0051)

*Bolded numerical entries are the Brier scores for which the training and test data are identical.

**95% CI intervals for Brier Scores and differences are given in parentheses.

**Table 3:** The ethnicity of smoking and non-smoking subjects.

	Smoker	Control
Caucasian	351 (83%)	371 (88%)
African American	48 (11%)	11 (3%)
Asian	5 (1%)	24 (4%)
Other	17 (4%)	17 (4%)
Total	421	423

**Figure 4:**
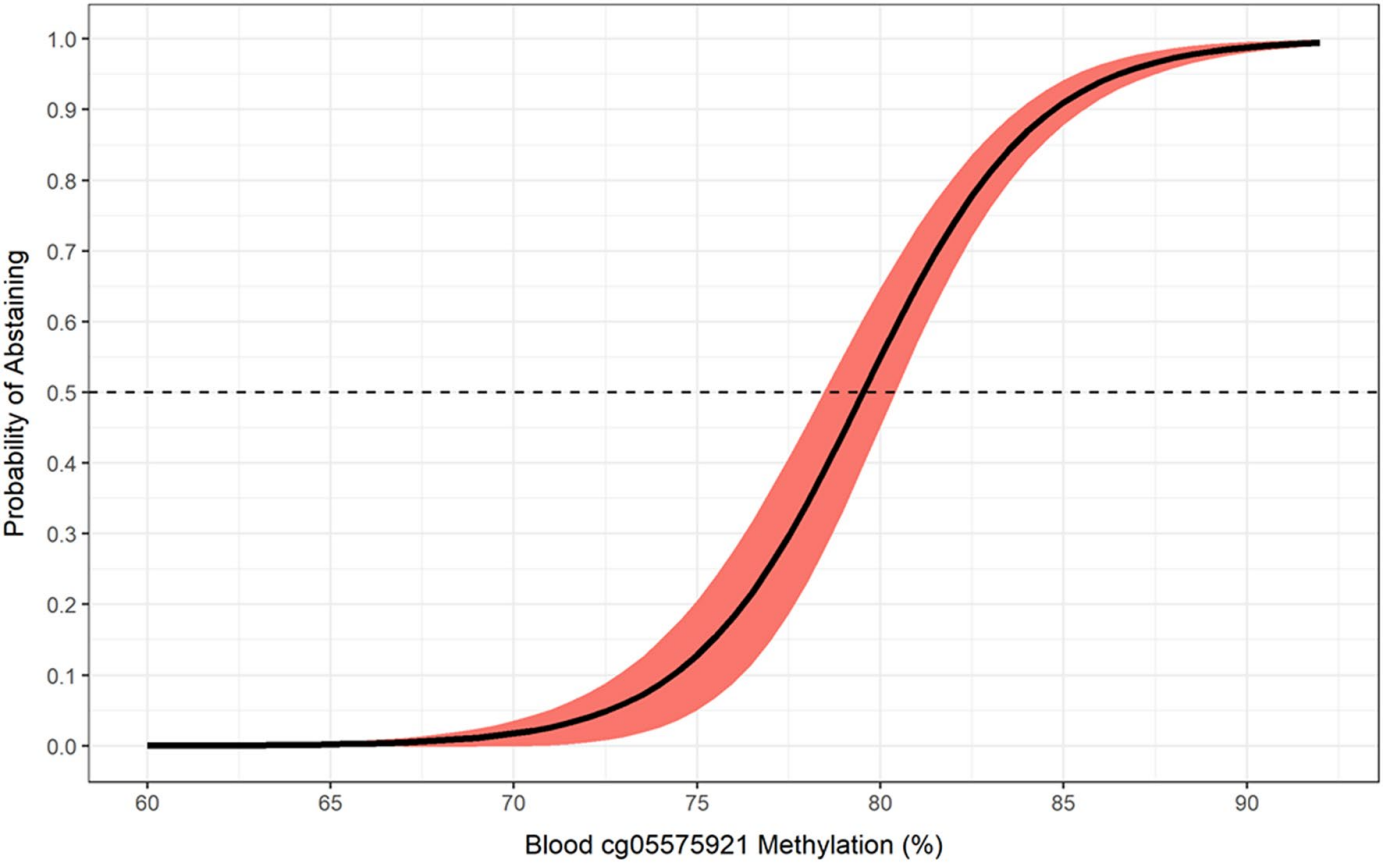
Predicted probability (95% confidence ribbon) of abstaining from smoking as a function of whole blood cg05575921 methylation percentage.

Table 4 provides the parameters for the final Model 1. The area under the curve (AUC) is 0.984 (95% CI 0.975–0.991). Figure 4 illustrates the relationship between WB cg05575921 methylation level with the probability that they are a smoker in this cohort of subjects of whom almost exactly 50% reported smoking. Using the base cg05575921 model, there is a steady increase in probability of smoking as DNAm decreases from 90 to 83% (i.e. < 10% probability with 85% DNAm). Next, a sharp ~ 10% increase in probability is observed per 1% decrease in DNAm between 81 and 79% methylation (i.e., 56% probability with 79% DNAm). Then, the rate of probability levels out to 99% probability when DNAm is less than 68%.

**Table 4:** Model 1 (WB DNA) Parameter estimates and calibration statistics with 95% CI bounds for the pooled data (top), ALC2A (middle), and SFW (bottom).

Model	Effect/Statistic	Estimate	Lwr	Upr
Model 1 Pooled (*N* = 779)	Intercept	− 33.683	− 40.3087	− 27.0573
	WB cg05575921	0.4235	0.3434	0.5036
	*Brier Score*	0.0433	0.033	0.0537
	*AUC*	0.984	0.9753	0.991
Model 1 ALC2A (*N* = 260)	Intercept	− 34.3824	− 50.3077	− 23.4395
	WB cg05575921	0.4370	0.3045	0.6319
	*Brier Score*	0.0405	0.0221	0.0590
	*AUC*	0.9815	0.9610	0.9953
Model 1 SFW (*N* = 351)	Intercept	− 29.9781	− 41.4922	− 22.5292
	WB cg05575921	0.3838	0.2940	0.5165
	*Brier Score*	0.0466	0.0304	0.0629
	*AUC*	0.9699	0.9439	0.9883

### Saliva DNA analyses

**Figure 5:**
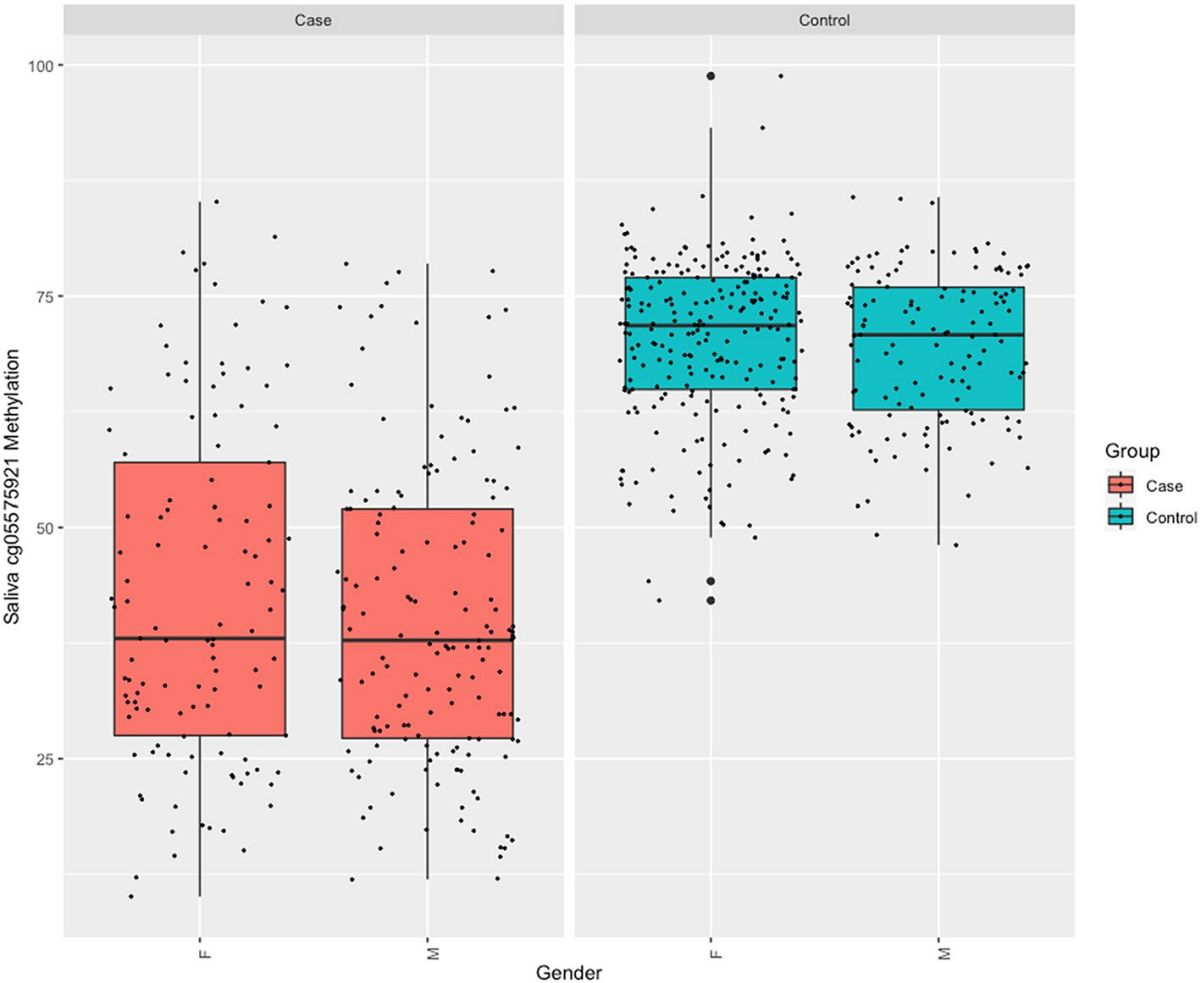
Box plots of the distribution of cg05575921 values in saliva samples from case (n = 266) and control (n = 363) subjects as a function of gender (M = male, F = female).

**Figure 6:**
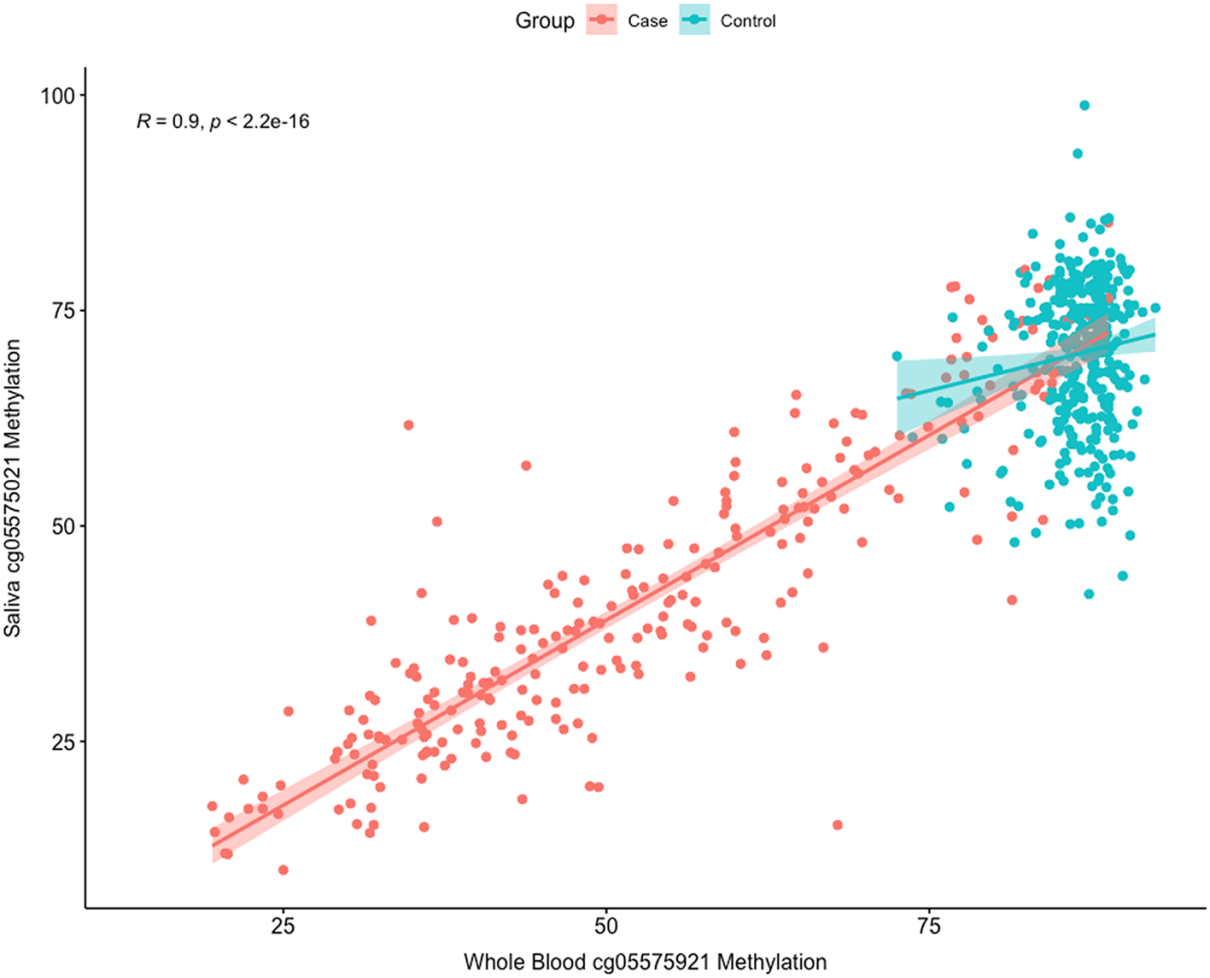
The relationship of WB cg05575921 methylation to saliva cg05575921 methylation. The salmon colored line represents the best fit line for the smoking subjects while the blue line represents the best fit line for the non-smoking subjects.

As a first step, we analyzed the relationship of cg05575921 DNAm in WB DNA to that in saliva DNA in 629 paired samples (266 cases, 363 controls). Figure 5 illustrates the distribution of uncorrected cg05575921 methylation as a function of case or control and stratified by gender. As compared to WB cg05575921 methylation, the range and distribution of saliva DNA cg05575921 methylation values is visibly larger and broader in the control subjects. Figure 6 illustrates the uncorrected correlation of the paired samples. In general, the cg05575921 DNAm values between the two tissues were highly correlated in the pooled cohorts (*p* < 2.2e−16, r = 0.9) and the individual cohorts: SFW (*p* < 2.2e−16, r = 0.85), ALC2A (*p* < 2.2e−16, r = 0.95), and NC (*p* < 2.2e−16, r = 0.83). Please note that the SCT cohort did not have saliva DNA specimens.

Next, we examined the ability of uncorrected cg05575921 methylation to predict average daily cigarette consumption within the past month and pack year consumption. Using a smooth cubic spline, a similar dose response is observed in uncorrected saliva values to both daily cigarette (Supplement Fig. 3; *p* < 0.0001, R^2^ = 0.5340) and pack year consumption (*p* < 0.0001, R^2^ = 0.4352) to that observed in WB methylation.

**Figure 7:**
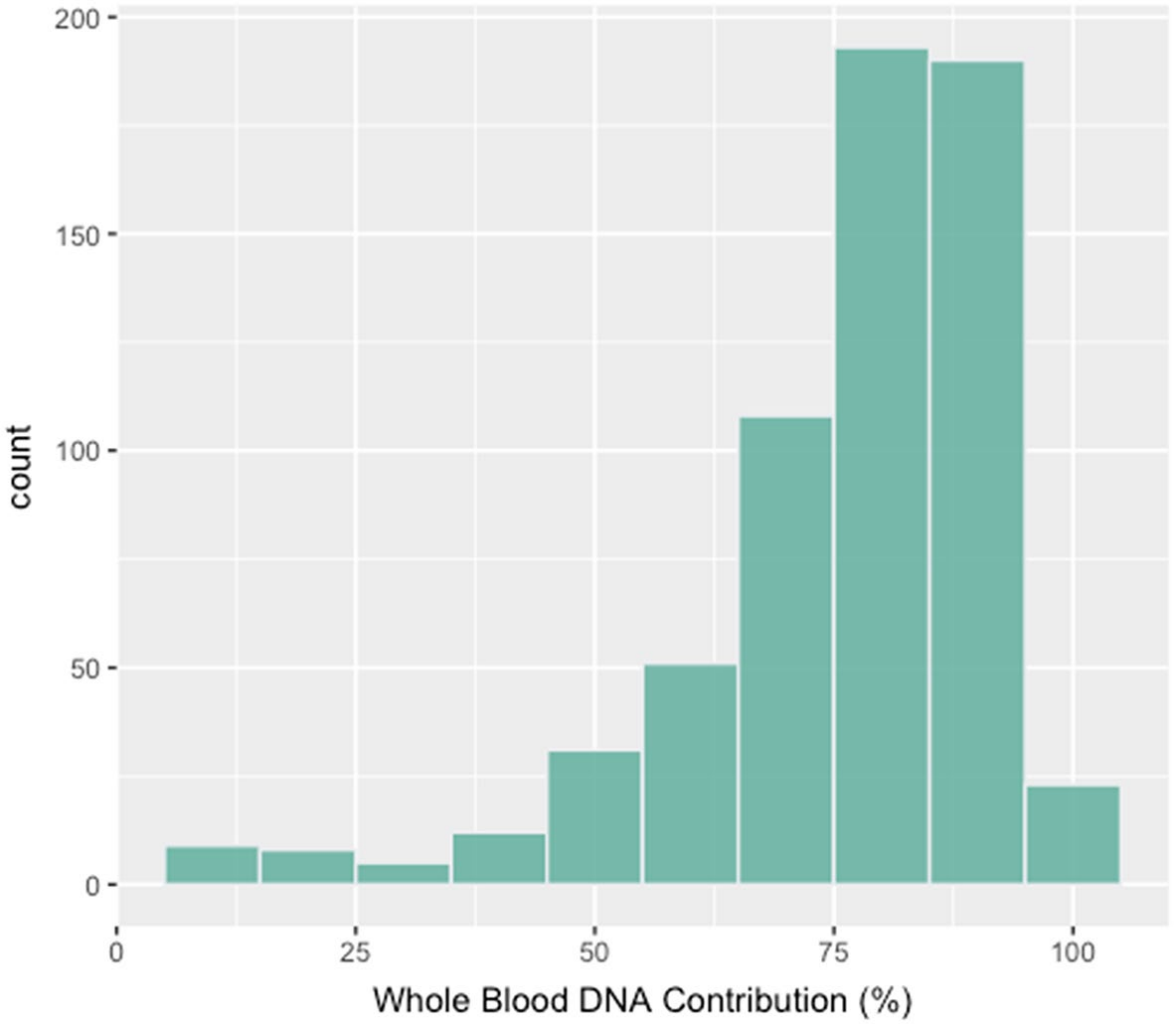
The WB contribution to the total human DNA component of saliva DNA as determined using the DMR11 assay (n = 601).

The imperfect correlation of WB cg05575921 methylation in WB to that of saliva is due in part to the cellular heterogeneity found in saliva. To better understand this heterogeneity, we used the DMR11 assay to impute the ratio of buccal and leukocytes in each saliva sample (Fig. 7). The average contribution of leukocyte DNA to the total human DNA in saliva was 75.9% ± 16.7% (n = 603).

#### Prediction capacity

In our final set of analyses, we attempted to predict smoking status using methylation data only from the saliva samples. Table 5 shows the Brier score results for Model 2. The values are larger than for Model 1 indicating generally lower prediction accuracy for saliva assessments. The first row shows that training on ALC2A and testing on SFW produces a larger Brier score than testing on ALC2A itself, but a smaller value when testing on NC. The lower portion shows that these differences are not statistically significant (0 is not in the CI). In contrast, training on SFW and testing on ALC2A or NC was not statistically different than testing on SFW itself. The AUC using saliva DNA was 0.939 (95% CI 0.915 to 0.962) was lower than that for WB DNA.

**Table 5:** Model 2 (Saliva DNA) Brier scores and Brier scores differences for the test and training data sets.

Train	Test			
	ALC2A	SFW	NC	SCT
**Brier score**				
ALC2A	**0.0638 (0.0608, 0.0739)**	0.1031 (0.0704, 0.1652)	0.0424 (0.0239, 0.1120)	–
SFW	0.0713 (0.0637, 0.0811)	**0.0665 (0.0641, 0.0715)**	0.0944 (0.0641, 0.1448)	–
**Difference**				
ALC2A	–	− 0.0393 (− 0.0999, − 0.0044)	0.0214 (− 0.0492, 0.0414)	–
SFW	− 0.0048 (− 0.0157, 0.0039)	–	− 0.0280 (− 0.0771, 0.0035)	–

*Bolded numerical entries are the Brier scores for which the training and test data are identical.

**95% CI intervals for Brier Scores and differences are given in parentheses.

## Discussion

In this communication, we present the analyses of data from four prior studies in order provide a more complete picture of the ability of a MSdPCR assay to assess cigarette consumption and classify subjects as smokers or non-smokers using DNA from whole blood or saliva. These findings show that the assay is a robust method for assessing smoking status and intensity. However, before considering these findings, certain limitations should be noted. First, although the assay is commercially available, the data analyzed are only from our laboratory group. Second, even though there is considerable representation of non-White ethnicities, all of the subjects are from the Midwest region of the United States which has excellent air quality. If environmental influences such as air pollution substantially affect the methylation set point, the application of this assay to regions of the world with significant exposure may be affected. Third, this manuscript does not address the issue of classifying former smokers. This limitation is discussed in depth below.

Perhaps the interesting finding of the study is that methylation at this locus can be used “straight up”, without factoring in age or sex, to predict smoking status. This appears to be a fortunate finding because many other CpG loci experience “epigenetic drift”, a phenomenon that has been harvested to construct indices of epigenetic aging^43–45^. Establishing this lack of variability of the set point at this locus point is critical to the field because smoking is a major driver of accelerated aging with cg05575921 methylation being associated with methylation status at least 38% of 513 markers in one popular EA index^46^. Indeed, if EA indices are to be used in guiding healthcare decisions, it is essential that they be interpretable in a clinically meaningful and reliable manner. In this regard, estimates of smoking status can be derived from some EA indices^47^. But to the best of our knowledge, the inter-assay variability of these EA assessments has not been published nor are the methods through they impute smoking independently verified. In contrast, the inter-assay variability of this reference free MSdPCR method used herein is established (0.8%) with the current data giving a clear basis for interpreting results (e.g. cigarettes per day)^23–25^.

Establishing the methylation set point in non-smokers was not a trivial undertaking. Our efforts have had to address a number of problems including two key barriers in defining the set point of cg05575921 methylation in non-smoking individuals. The first barrier was the problem of subjects presenting themselves as non-smokers when they are in fact smoking. In our prior efforts, we have noted that between 3 and 5% of individuals presenting themselves as lifetime non-users of any tobacco or cannabis product have significant serum levels of cotinine or cannabinoids^26,30^. The rate of misrepresentation is even higher in some of our longitudinal studies with the rate of former smokers misrepresenting their past smoking being completely unknown^13^. The second challenge to establishing a set point is variability in air quality. Studies from Taiwan have shown that air quality can have a small, but significant effect on cg05574921 levels^48^. However, the levels of air pollution in Taiwan are relatively low and fortunately, the level of PAH in the Midwestern United States are relatively low^49^. But in other parts of the world, in particular those which rely on wood or coal fired power production, the exposure could be considerable^50^. Occupational exposure may magnify this problem. We note prior publications noting considerable levels of PAH exposure in steel workers^51^. Together with other problems, these issues have made establishing the non-smoking threshold of cg05575921 methylation a challenging proposition.

To address these challenges and establish the methylation set points of cg05575921 methylation, we have surveyed populations with low likelihood of significant underreporting to get a better understanding of the assay set point in various groups. The most notable effort prior to this communication has been the Healthy Iowan Study^22^. In that study, on the presumption that most high school sophomores will not yet have started smoking, we enrolled 15 year-old high school students and followed them for two years, drawing their blood and measuring their CO levels at three points of time. In the 364 subjects who denied any type of smoking and had negative cotinine and cannabinoid levels, the average level of cg05575921 methylation at age 15 using this assay was 86.8% ± 3.2^22^. That is very similar to the 86.6% ± 2.9 found in these 423 biochemically screened adults and strongly argues against any significant age dependent changes in cg05575921 methylation in adult subjects.

For those conducting clinical studies, the most interesting results in these analyses may those for the saliva specimens. In brief, we show that consistent with prior analyses by ourselves and others, approximately 76% of the human portion of saliva DNA is derived from white blood cells and that the use of the DMR11 assay improves prediction^26,52^. When the National Institutes of Health (NIH) funded the commercialization projects supporting the development of this assay were initiated, the initial, explicit purpose was to develop a blood-based assay compatible with existing sample flow in hospital laboratories. Since that time, the COVID19 pandemic and the rise of telemedicine (TM) approaches for provision of patient care have led to the need for assay systems that do not require blood draws and are not temperature sensitive. As a result, subsequent NIH commercial awards (R43AA027423; Philibert PI) were made to develop an assay such as DMR11 and the previously described DMR16 assay, which are capable of correcting for the heterogeneity of saliva^26,35,53^ and can use DNA from commercially available sampling kits, such as those provided by DNA Genotek (www.dnagenotek.com)^54^. Using these types of kits, providers such as Intelelabs (www.intelelabs.com) are already using TM to offer genetic testing. The current data strongly suggests that epigenetic testing for cg05575921 could also be conducted using these kits via standard TM approaches. If so, this would add yet another method through which clinicians could assess and monitor smoking cessation therapy.

Although it is clear that for classification purposes, cg05575921 information is sufficient, one question that these data do not address is whether the addition of genetic information may aid in understanding the relationship of smoking intensity to cg05575921. This is an important question. Nicotine, though addictive, has very few other adverse effects at pharmacologically relevant doses^55^. Instead, it is the other constituents in tobacco smoke, such as polyaromatic hydrocarbons, that cause smoking associated diseases^56^. Nevertheless, even though it is clear that we can precisely measure cg05575921 methylation levels and the basal set point appears to be reliably determined, the relationship between increasing cigarette consumption and decreasing cg05575921 levels is only modestly strong (R^2^ ~ 0.62). There are several reasons for this less than complete accounting for the variance in the relationship. First, subjects do not always accurately recall their cigarette consumption. Second, the binning of responses into easy to express units (e.g. “half-pack”) may contribute to the error. Finally, subjects vary in the number and size of puffs that they extract from each cigarette^57^. But another factor that may affect the dose response relationship is that genetic variation in the cytochromes regulated in part by AHRR may alter the magnitude of response^58^. If so, by defining cytochrome variation, it may be possible to devise new methods to induce the detoxification of PAH that can be used for preventing cancer in those who cannot avoid PAH exposure.

Finally, as noted in the limitations section, this analysis does not address the use of cg05575921 methylation to classify former smokers. Several studies have shown that cg05575921 methylation undergoes a slow reversion to the mean as a function of initial smoking intensity^7,8,28,59^. In brief, those studies show that the longer and more intensively one smokes, the longer it takes to revert back to baseline after quitting. But the number of subjects and the time periods examined in our studies and those of others are limited. As a consequence, we do not believe that there is a sufficient evidence base to guide prediction of when a former smoker will cross the 80% threshold associated with non-smoking status.

### Conclusions

In summary, in this joint analysis of samples of subjects from four rigorous studies, we show that cg05575921 methylation levels can be used to accurately classify subjects with respect to smoking and infer daily cigarette consumption using DNA from either whole blood or saliva. Critically, unlike methylation array approaches for assessing smoking, the MSdPCR method used in these assessments can be rapidly performed by any of the thousands of laboratories with digital PCR machines with the results being expressed as β or % methylation values that are directly interpretable. The resulting methylation levels will be of potentially informative for clinicians assessing patients for need for low dose CT lung screening^60^ or for guiding smoking cessation therapy^28^. Efforts to make this assay more available to clinicians through independent regional facilities are in progress.

## Supplementary Information

Supplementary Figures.

Supplementary Information.

## Data Availability

The data used in the analyses can be obtained upon reasonable request to the principle investigators for each study cohort.
