# Supplementary material for: The relationship of smoking to cg05575921 methylation in blood and saliva DNA samples from several studies
Source: Sci Rep. 2021 Nov 3;11:21627. doi: 10.1038/s41598-021-01088-7 (PMC8566492; doi:10.1038/s41598-021-01088-7)
Supplement: Supplementary file 1 — Supplementary Figures. [file 41598_2021_1088_MOESM1_ESM.docx]

**Supplemental Figures**

**
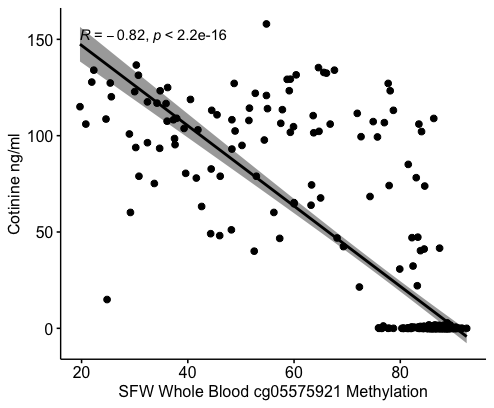
**

Supplemental Figure 1. The relationship between serum cotinine levels and WB cg05575921 levels (%) in the SFW cohort ( r = - 0.82, p < 2.2e-16).

**
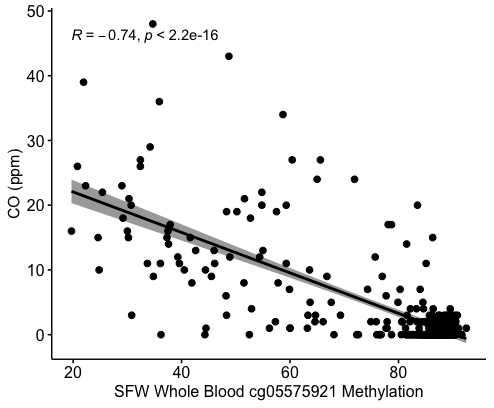
**

Supplemental Figure 2. The relationship between exhaled carbon monoxide levels and WB cg05575921 levels (%) in the SFW cohort (r = - 0.74, p < 2.2e-16).

**
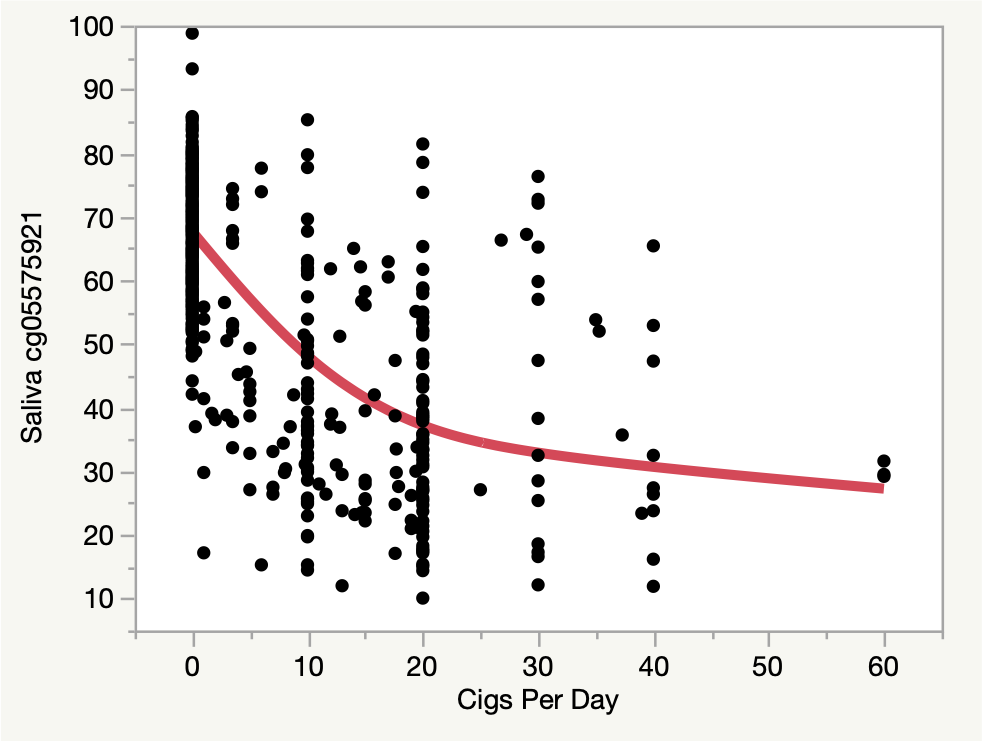
**

Supplemental Figure 3. The relationship of saliva cg05575921 methylation to self-reported daily cigarette consumption. The line in red represent a smoothing spline fit with a lambda of 100000 (p < 0.0001, R^2^ = 0.5340).
