# Supplementary material for: The relationship of smoking to cg05575921 methylation in blood and saliva DNA samples from several studies
Source: Sci Rep. 2021 Nov 3;11:21627. doi: 10.1038/s41598-021-01088-7 (PMC8566492; doi:10.1038/s41598-021-01088-7)
Supplement: Supplementary file 2 — Supplementary Information. [file 41598_2021_1088_MOESM2_ESM.docx]

**Supplementary Methods**

**Full Description of the Four Cohorts**

The data for these analyses are derived from subjects included in four previously published studies of smoking or alcohol use [1-4]. In each of these studies, methylation status at cg05575921 status in WB or saliva DNA was determined using MSdPCR and the smoking status of each non-smoking control was verified using serum cotinine assessments and carbon monoxide assessments as previously described [1, 4]. All procedures were performed in accordance with the Declaration of Helenski with the individual institutional approvals noted separately for each cohort. All subjects provided written consent for the procedures for each of the follow studies.

The Nicotine Cessation (NC) cohort subjects were collected under the auspices of R44CA213507, a National Institutes of Health (NIH) funded project whose goal was to help define the reversion curve of cg05575921 in response to smoking cessation [1]. In brief, subjects from an Iowa community substance use treatment who stated an interest in smoking cessation, reported smoking at least 8 cigarettes per day and had an expired carbon monoxide level of 8 ppm or greater, were eligible for inclusion for this longitudinal study of smoking cessation. After consent, adult subjects were interviewed with a modified version of the Semi-Structured Assessment for the Genetic of Alcoholism and the Substance Use Questionnaire [5, 6]. Then, both WB and saliva specimens were collected to provide biomaterials for serum and DNA preparation. A total of 114 subjects enrolled in the study and completed the intake visit. All study procedures were approved by the Western Institutional Review Board (WIRB20162083).

The Alcohol Cessation (ALC2A) subjects were collected under the auspices of R44AA022041, a NIH funded project whose goal was to develop a blood-based test for heavy alcohol consumption [2]. In this protocol, two distinct groups of subjects were collected. The first group consisted of self-reported smokers who were admitted to one of three Iowa alcohol treatment centers for the treatment of severe alcohol use disorder in the context of current intoxication. After detoxifying, subjects who were interested participating were approached by staff members and educated on the protocol, and if still interested, enrolled in the study. Although it was not necessary to be a smoker to be included in the alcohol study, only the data from the self-reported smokers were included in this study (127 of 143 total). The second group of subjects (i.e. controls) collected under this award were individuals solicited from the University of Iowa community who denied any history of substance abuse and reported at least one year of abstinence from alcohol. After informed consent was received, both sets of subjects were then were interviewed with a modified version of the Semi-Structured Assessment for the Genetic of Alcoholism and the Substance Use Questionnaire, with both saliva and blood collected for biomaterial preparation [5, 6]. The procedures used in this study were also approved by the Western Institutional Review Board (WIRB 20160135).

The Smoke Free World (SFW) cohort was collected under an award to Behavioral Diagnostics from the SFW Foundation with all clinical collections proceeding through subcontracts with the University of Iowa and Des Moines University. All procedures in this study were approved by the University of Iowa IRB (IRB 201905678). In this protocol, subjects who either denied lifetime use of tobacco products or reported the current use of cigarettes, vaping devices or smokeless tobacco were invited to participate in study whose purpose designed to test whether DNA methylation could be used to differentiate the use of non-combustible use of nicotine containing products from smoking [4]. After providing informed consent, adult subjects were administered a 320 item REDCap® interview that assessed nicotine product use history, then phlebotomized and saliva sampled to provide biomaterials for the current study [4, 7]. Although this project also recruited those who reported vaping or use of smokeless tobacco, only the data from the smoking subjects and the lifetime non-smoking controls were included in this study.

The Smoking Computerized Tomography (SCT) cohort was collected under a NIH award to the University of Iowa whose purpose was to understand the relationship of sildenafil treatment to pulmonary inflammation in subjects undergoing smoking cessation therapy [3]. In this protocol, which was approved by the University of Iowa IRB (IRB201706713), subjects who smoked at least 10 cigarettes per day and had a 5-pack year history of smoking were invited to complete a REDCap® administered prescreening interview to assess smoking history and study eligibility. After receiving informed consent, subjects were phlebotomized to provide blood for DNA for epigenetic analysis and serum.

Exclusion Criteria: Because smoking is not legal for those under the age of 18 and the focus of the study was classifying adult smokers, subjects under the age of 18 were excluded from the study. Subjects actively under the influence of substances or otherwise not capable of giving informed consent were excluded from the studies.

1. Philibert R, Dogan M, Beach SRH, Mills JA, Long JD: **AHRR methylation predicts smoking status and smoking intensity in both saliva and blood DNA**. *Am J of Genet* 2019, **183**(1):51-60.

2. Philibert R, Miller S, Noel A, Dawes K, Papworth E, Black DW, Beach SRH, Long JD, Mills JA, Dogan M: **A Four Marker Digital PCR Toolkit for Detecting Heavy Alcohol Consumption and the Effectiveness of Its Treatment**. *Journal of Insurance Medicine* 2019, **48**(1):90-102.

3. Philibert R, Mills JA, Long JD, Salisbury SE, Comellas A, Gerke A, Dawes K, Vander Weg M, Hoffman EA: **The Reversion of cg05575921 Methylation in Smoking Cessation: A Potential Tool for Incentivizing Healthy Aging**. *Genes* 2020, **11**(12):1415.

4. Andersen A, Reimer R, Dawes K, Becker A, Hutchens N, Miller S, Dogan M, Hundley B, A Mills J, D Long J *et al*: **DNA methylation differentiates smoking from vaping and non-combustible tobacco use**. *Epigenetics* 2021:1-13.

5. Philibert R, Penaluna B, White T, Shires S, Gunter T, Liesveld J, Erwin C, Hollenbeck N, Osborn T: **A pilot examination of the genome-wide DNA methylation signatures of subjects entering and exiting short-term alcohol dependence treatment programs**. *Epigenetics* 2014, **9**(9):1212-1219.

6. Bucholz KK, Cadoret R, Cloninger CR, Dinwiddie SH, Hesselbrock VM, Nurnberger JI, Jr., Reich T, Schmidt I, Schuckit MA: **A new, semi-structured psychiatric interview for use in genetic linkage studies: a report on the reliability of the SSAGA**. *J Stud Alcohol* 1994, **55**(2):149-158.

7. Harris PA, Taylor R, Minor BL, Elliott V, Fernandez M, O'Neal L, McLeod L, Delacqua G, Delacqua F, Kirby J: **The REDCap consortium: Building an international community of software platform partners**. *Journal of biomedical informatics* 2019, **95**:103208.
